# Experimental investigation into shear behavior of loess-red clay composite interface under low-temperature environment

**DOI:** 10.1038/s41598-025-31333-2

**Published:** 2025-12-09

**Authors:** Jingjing Nan, Bin Chen, Qiang Sun, Jianbing Peng, Yuan Xin, Pengda Ma, Qiang Li

**Affiliations:** 1 College of Geology and Environment, Xi’an University of Science and Technology, Xi’an, 710054 Shaanxi China; 2 Postdoctoral Research Station of Geological Resource and Geological Engineering, Xi’an University of Science and Technology, Xi’an, 710054Shaanxi China; 3 Shaanxi Provincial Key Laboratory of Geological Support for Coal Green Exploitation, Xi’an, 710054 Shaanxi China; 4 Department of Geological engineering, Chang’an University, Xi’an, 710054 Shaanxi China; 5 China University of Mining and Technology, Beijing, 100080 China

**Keywords:** Loess, Loess–red clay composite interface, Moisture content, Temperature, Shear behavior, Engineering, Environmental sciences, Natural hazards

## Abstract

The shear behavior of the double-layer heterogeneous interface between loess and red clay is crucial for better understanding the formation and evolution mechanism of loess–red clay interface landslides in the Loess Plateau. However, the effects of low-temperature environment on the shear behavior of loess–red clay composite interface are given lesser attention. This study conducted freezing and direct shear tests on loess–red clay composites with different moisture contents to investigate the effects of moisture content and low temperature on the shear behavior of the interface. The results indicate that the shear strength of the interface decreases with increasing moisture content. Under high moisture contents (> 14%), the red clay tends to form aggregates, intensifying the double-layer heterogeneity and further decreasing shear strength. With decreasing temperature, the shear strength exhibits a three-stage trend of “increase (unfrozen stage (–2°C~ − 5°C))-decrease (transition zone (–5°C~ − 10°C))-increase (freezing stage (–10°C~ − 20°C))”, mainly attributed to water migration and water-ice phase transition evolution under different low-temperature environments. Considering the coupling effects of moisture content and temperature, the shear strength is divided into four intervals using a threshold of 14% moisture content (aggregation formation) and − 10 °C (freezing point). The average shear strength in each interval shows a negative correlation with moisture content, confirming that moisture content is the dominant factor affecting the shear strength of the interface. These findings can facilitate the disaster prevention and control of heterogeneous interface landslides and the security of major engineering construction in seasonal frost and permafrost loess areas.

## Introduction

The loess interface is a product of the formation and evolution of loess, playing a crucial role in controlling the geological structure and acting as a primary factor in loess disasters^1^. In the Loess Plateau of northwest China, a widespread layer of Tertiary red clay—commonly known as “Hipparion red clay” (hereafter referred to as “red clay”)—is deposited between the Quaternary loess and the underlying bedrock, forming a “double-layer heterogeneous soil” contact interface^1,2^. The loess and red clay differ significantly in their physical and mechanical properties^3–5^. Under external environmental factors such as water, stress, and temperature, shear deformation and failure are prone to occur along this interface^6^, which serves as a natural weak slip surface and plays a controlling role in slope evolution and stability^2,6^. Moreover, the average winter temperature in the Loess Plateau region is below − 3 °C, with extreme lows reaching − 28 °C. Seasonal frozen soils are widely distributed, and some areas even experience permafrost^7–10^. Under low-temperature conditions, the loess–red clay interface exhibits varying degrees of sliding risk. As the temperature drops, the soil becomes more brittle after freezing. During precipitation events, both the soil and the interface may soften, leading to a reduction in shear strength and increased deformation, thereby triggering slope instability and sliding^11^. These conditions often lead to various medium- and large-scale loess landslides, particularly of the loess–red clay contact type, with a higher frequency of occurrence during the thawing season from January to April, posing serious threats to infrastructure and human settlements^12–14^. Therefore, studying the shear behavior of the loess–red clay composite interface under different moisture contents in low-temperature environment is of great significance for understanding the catastrophic effects and prevention and control of heterogeneous interface landslides in seasonally and permanently frozen areas of the Loess Plateau. At the same time, it can also provide scientific references for engineering implementation and safe operation in loess areas.

The direct shear test simulates the interaction between overlying soil or rock masses by applying shear forces, and it effectively captures the interface shear deformation and failure processes that can lead to sliding^15,16^. At present, many researchers have conducted extensive studies on the shear behavior and influencing factors of various types of binary heterogeneous interfaces using direct shear tests^17–21^. For example, to investigate the interface strength between soil and concrete in permafrost regions, Liu et al.^22^ performed direct shear tests on frozen soil–concrete interfaces under low-temperature conditions. Their results indicated that normal stress and environmental temperature were the dominant factors affecting the interface shear strength, both showing a linear relationship with shear strength, while moisture content had little effect. Cen et al.^23^ studied the shear deformation and strength characteristics of interfaces between mixed soil–rock fill slopes and stepped bedrock surfaces. They found that increasing the proportion and size of rock blocks led to more pronounced fluctuations in the post-peak shear stress–displacement curves and expanded the shear failure zone network. Li et al.^24^ developed a large-scale direct shear apparatus to test the mechanical properties of soil–concrete interfaces under varying soil types, moisture contents, and interface-filling materials. The results showed that filling the sand–concrete interface with a thin layer of sandy soil reduced both the friction angle and cohesion, whereas filling with a thin layer of silt reduced the friction angle but increased cohesion. Yang et al.^17^ investigated the anisotropic shear behavior of loess–bedrock interfaces in Shanxi Province through indoor direct shear tests. Their findings demonstrated that the undulating nature of the shear surface was a major cause of anisotropy at the loess–bedrock interface. The shear characteristics of soil–geosynthetic interfaces are key factors influencing the load transfer mechanism. He et al.^18^ conducted direct shear tests on silty clay and composite geotextiles under freeze–thaw conditions using an enhanced temperature-controlled direct shear apparatus. The results showed that the interface shear strength initially increased and then decreased with the number of freeze–thaw cycles, and moisture content was identified as the primary factor affecting the interface shear strength. Previous studies have primarily focused on the shear properties of interfaces between soil and structures or soil and bedrock, mostly under positive temperature conditions. Although preliminary investigations have been conducted on the shear strength characteristics of the loess–red clay interface^25–29^, research on the effects of low-temperature freezing on the shear behavior of loess–red clay composite interface with varying moisture contents remains limited and requires further exploration.

Therefore, this study focuses on the shear behavior of loess–red clay composite interface under low-temperature conditions. By conducting freezing and direct shear tests on loess–red clay composites with different moisture contents (8%–20%) at different temperatures (–2 °C to − 20 °C), the changes in the shear behavior of the interface under different moisture and temperature conditions were analyzed. The aim is to reveal the mechanisms by which moisture content, low temperature, and their coupling effects influence the shear strength of the loess–red clay composite. The experimental results provide valuable data for understanding the shear behavior of loess–red clay composites in cold environments, and provide a theoretical basis for the formation and evolution mechanism of loess–red clay interface landslides, major engineering construction, and disaster prevention and reduction in the Loess Plateau.

## Materials and methods

### Test material and sample Preparation

**Fig. 1:**
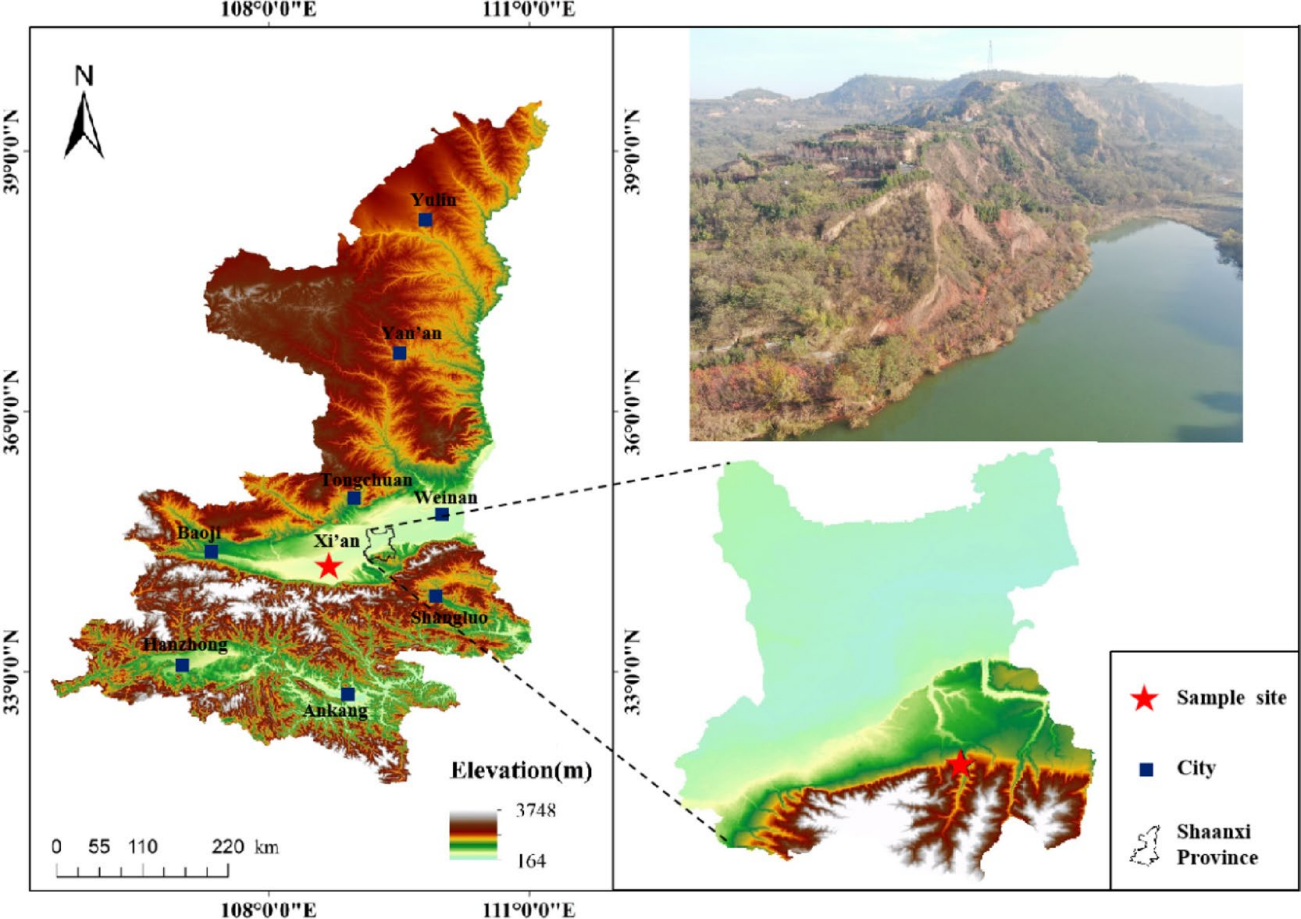
Location of the sampling site.

**Fig. 2:**
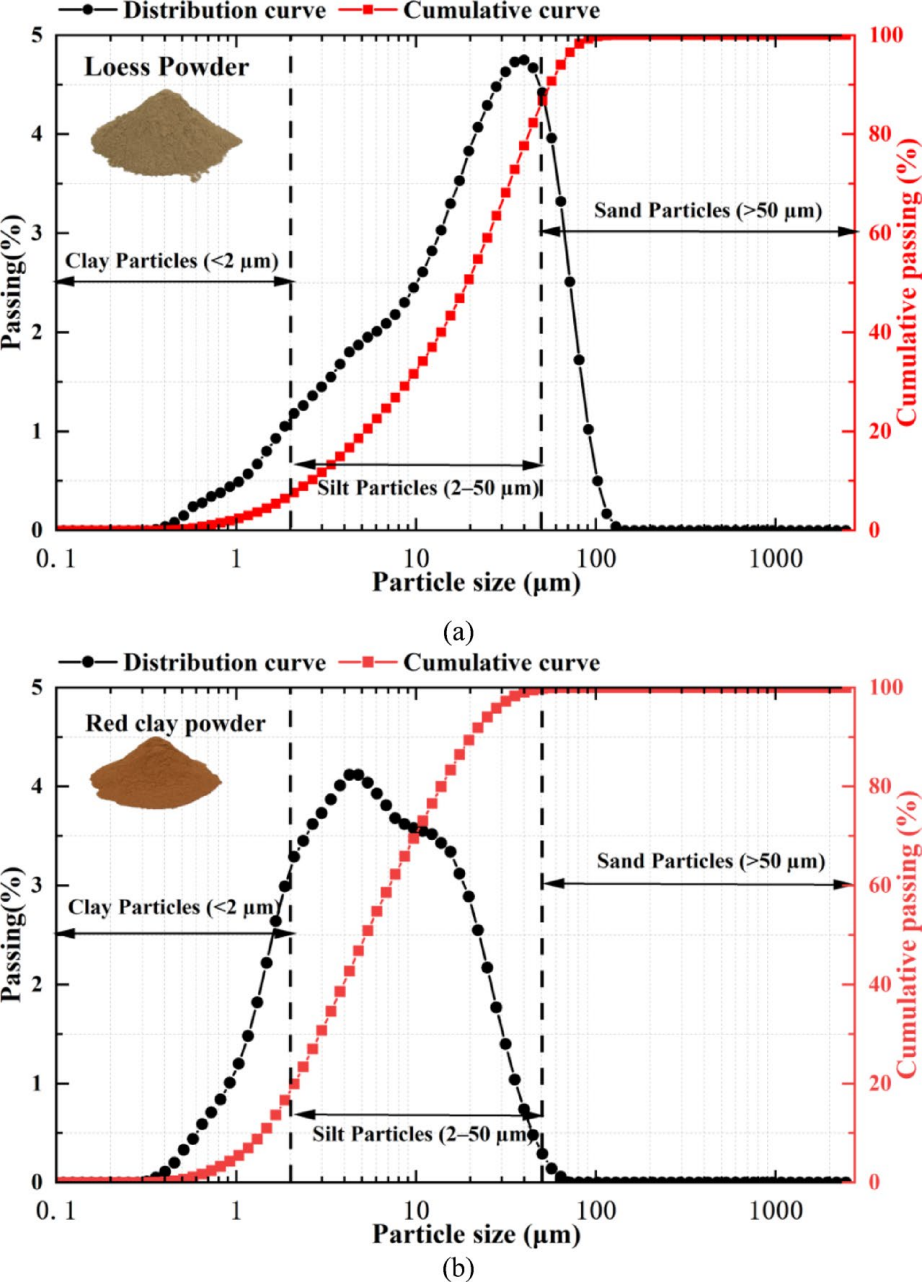
Particle size distribution curve of the experimental soil. (**a**) Particle size distribution curve of loess, (**b**) Particle size distribution curve of red clay.

The study area is located in Lintong District, Xi’an City, Shaanxi Province (Fig. 1), where the loess–red clay stratigraphic combination is commonly found. The overlying loess is characterized by high porosity, well-developed joints and fissures, loose structure, and strong permeability, while the underlying red clay has a high clay content with poor permeability and low resistance to softening. These contrasting properties often lead to water accumulation at the interface and slope toe, which, under varying temperature conditions, can trigger slope deformation, failure, and interface shear sliding. The test soil samples were collected from a single stratigraphic profile in the study area, including both the overlying loess and underlying red clay, at a depth of 10 m. After sampling, the specimens were sealed and transported to the laboratory for subsequent tests. The basic physical properties of the test materials are listed in Table 1. The natural moisture contents of the loess and red clay were 5.59% and 6.44%, respectively; their natural densities were 1.70 g/cm³ and 1.76 g/cm³; and their specific gravities were 2.70 and 2.72, respectively. Particle size distribution tests were conducted using the Bettersize2600 laser particle size analyzer (wet method). As shown in Fig. 2, the loess consisted of 7.65% clay, 79.11% silt, and 13.24% sand, while the red clay consisted of 19.93% clay, 79.87% silt, and 0.20% sand.

**Table 1:** Basic physical properties of the tested soils.

Property	w_*n*_/%	ρ/ g·cm^− 3^	G_s_	ρ_d_/ g·cm^− 3^	w_Sat_/%	W_*P*_	W_L_	I_*P*_
Loess	5.59	1.70	2.70	1.61	25.1	19.2	27.1	7.9
Red clay	6.44	1.76	2.72	1.65	23.8	18.5	29.7	11.2

Basic physical properties of the tested soils. ***w***_***n***_ represents natural moisture content; ***ρ*** represents natural density; ***G***_***s***_ represents specific gravity of soil particles; ***ρ***_***d***_ represents dry density; ***w***_***Sat***_ represents saturated water content; ***W***_***P***_ represents plastic limit; ***W***_***L***_ represents liquid limit; ***I***_***P***_ represents plasticity index.

**Fig. 3:**
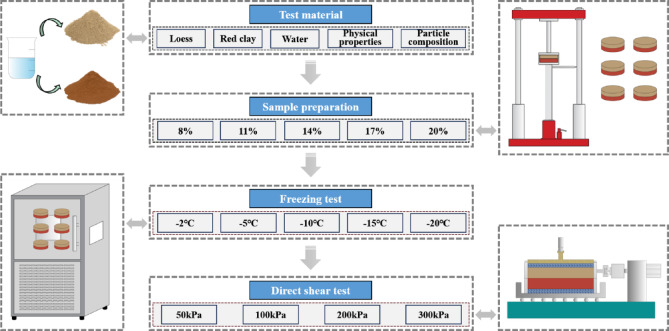
Test procedure.

The loess and red clay blocks collected from the field were oven-dried, crushed, and sieved to prepare the base materials. The moisture content was adjusted between the natural and saturated levels (Table 1), with five intermediate values selected: 8%, 11%, 14%, 17%, and 20%. The specimens were statically compacted in layers using a hydraulic jack, ensuring that both the loess and the red clay reached a dry density of 1.60 g/cm³, forming disc-shaped, ring-type samples. After demolding, each specimen measured φ61.8 mm × 20 mm, consisting of a 10 mm loess layer and a 10 mm red clay layer. The prepared specimens were wrapped in plastic film, labeled, and placed in a low-temperature chamber for 72 h prior to testing (Fig. 3). This standardized sample preparation procedure ensured structural consistency and uniform performance among parallel specimens. More detailed information about sample preparation can be obtained from the Chen et al.^6^。.

### Freezing test

The freezing tests were conducted using the Danfu TMS9018-1000 environmental resource freeze–thaw simulation system to replicate various low-temperature freezing conditions. This equipment can simulate environmental temperatures ranging from − 35 °C to 60 °C, with sample temperature control from − 50 °C to 60 °C. It is capable of year-round continuous operation to reproduce seasonal climate variations and is characterized by high precision, excellent stability, and operational flexibility.

The prepared loess–red clay ring samples with different moisture contents were wrapped in plastic film and placed in a sealed freezing chamber for 72 h to ensure complete freezing. Based on previous studies and the climatic and hydrological conditions of the study area and considering the applicability and feasibility of laboratory testing, the freezing temperatures were set at − 2 °C, − 5 °C, − 10 °C, − 15 °C, and − 20 °C. After freezing, the specimens were removed, photographed, and weighed.

### Direct shear test

Direct shear tests were conducted using a ZLB-1 triaxial rheological direct shear apparatus to investigate the shear mechanical behavior of the loess–red clay composite interface. This apparatus allows application of normal and shear stresses ranging from 0 to 600 kPa, with an accuracy of ± 1%, 100% humidity, and a power supply of 220 V ± 10%, 50 Hz.

The loess–red clay specimens with different moisture contents, after 72 h of freezing, were subjected to direct shear tests under normal stresses of 50 kPa, 100 kPa, 200 kPa, and 300 kPa. To ensure the specimen temperature, the shear box was placed in a low-temperature chamber for one hour before the specimens were installed for direct shear testing. After positioning the specimen, vertical pressure was applied for consolidation. When the vertical pressure reached the target normal stress, the consolidation amount was recorded. Consolidation was considered complete when the consolidation rate decreased to less than 0.005 mm/h. During shearing, the upper shear box was kept fixed, while the horizontal loading device pushed the lower shear box from the left. The tests were performed using a consolidated quick shear method with a shear rate of 0.8 mm/min. The test was terminated when the horizontal sensor detected a shear displacement of 18 mm. A total of 132 specimens, including supplementary samples, were used in this study.

## Results

### Shear Stress-Strain relationship

**Fig. 4:**
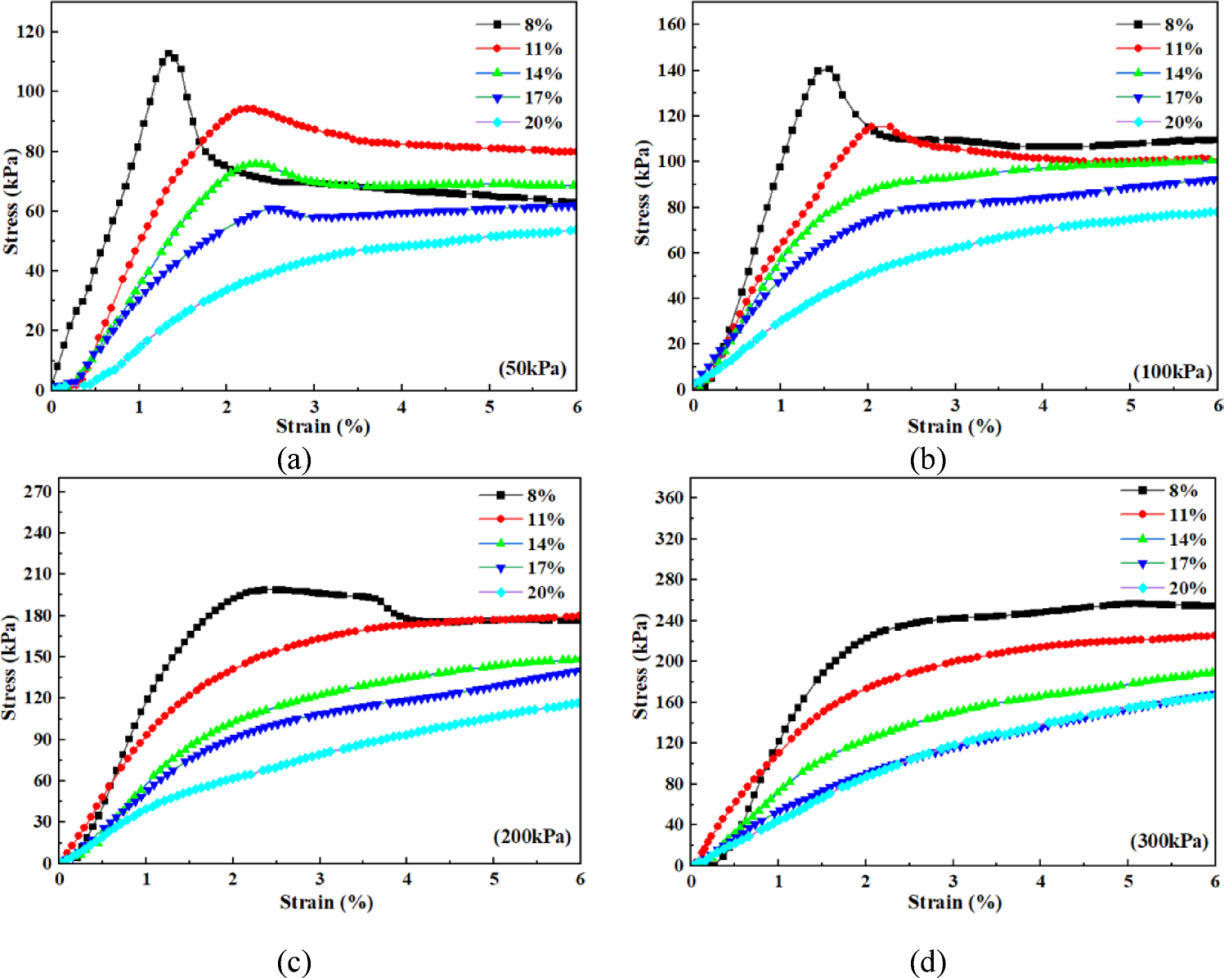
Shear stress-strain curves under different moisture contents at -10℃. (**a**) Stress-strain curves at different moisture contents under 50 kPa, (**b**) Stress-strain curves at different moisture contents under 100 kPa, (**c**) Stress-strain curves at different moisture contents under 200 kPa, (**d**) Stress-strain curves at different moisture contents under 300 kPa.

**Fig. 5:**
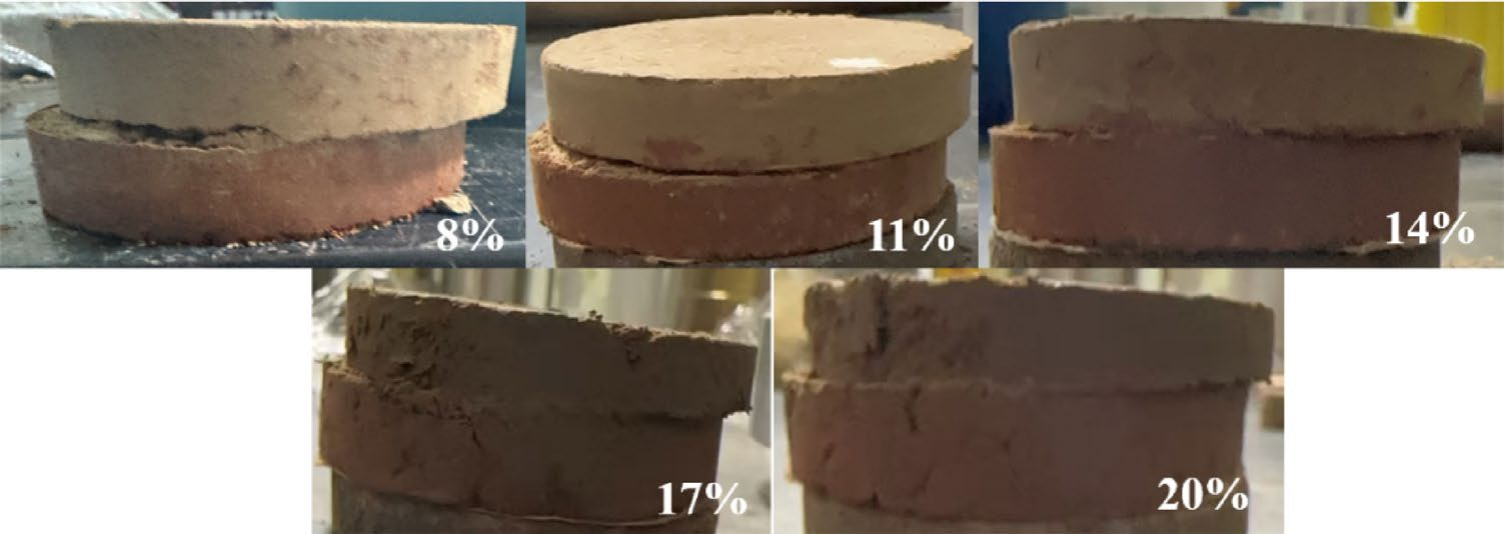
The state of the specimen after shearing.

Figure 4 illustrates the shear stress-strain curves of the loess–red clay composite under different normal stresses (50 kPa, 100 kPa, 200 kPa, and 300 kPa) at -10 °C, considering varying moisture contents. When the normal stress is 50 kPa (Fig. 4a), the shear stress-strain curve of the 8% moisture content sample initially rises, then declines, and finally stabilizes, exhibiting shear softening behavior. The peak shear strength is 112.8 kPa, corresponding to a peak strain of 1.3%. As the moisture content increases, the peak stress decreases while the corresponding strain increases. The stress-strain curve also transitions toward shear hardening, where the curve first rises monotonically to a higher value (inflection point) and then increases gradually or remains stable. For instance, at 11% moisture content, the peak shear strength is 94.3 kPa, marking a 16.4% decrease. At 14% and 17% moisture contents, the peak values are 75.9 kPa and 62.1 kPa, decreasing by 19.5% and 18.1%, respectively. When the moisture content reaches 20%, the curve fully exhibits shear hardening, and the stable stress at 6% strain is taken as the shear strength, which is 53.9 kPa–a 13.2% reduction. Under a normal stress of 100 kPa, samples with 8% and 11% moisture contents show strain-softening behavior, while those with 14% to 20% moisture contents exhibit shear hardening. The peak or stable shear strength of all samples exceeds 50 kPa, and as the moisture content increases, the inflection point of the curve appears later (corresponding to a higher strain). At a normal stress of 200 kPa, only the 8% moisture content sample exhibits strain softening, while samples with 11% to 20% moisture contents display shear hardening. The shear strength of all samples exceeds 100 kPa. When the normal stress is 300 kPa (Fig. 4d), the stress-strain curves for all moisture content conditions exhibit strain hardening behavior, with stable shear strengths exceeding 150 kPa. Overall, as moisture content and normal stress increase, the shear stress-strain curves transition from strain softening to strain hardening. The strain corresponding to peak or stable shear strength also increases, indicating an enhanced strain-hardening effect. In addition, it was observed that as the water content increases, the state of the sample after shearing transitions from a separated state to an intact, non-separated state (Fig. 5).

**Fig. 6:**
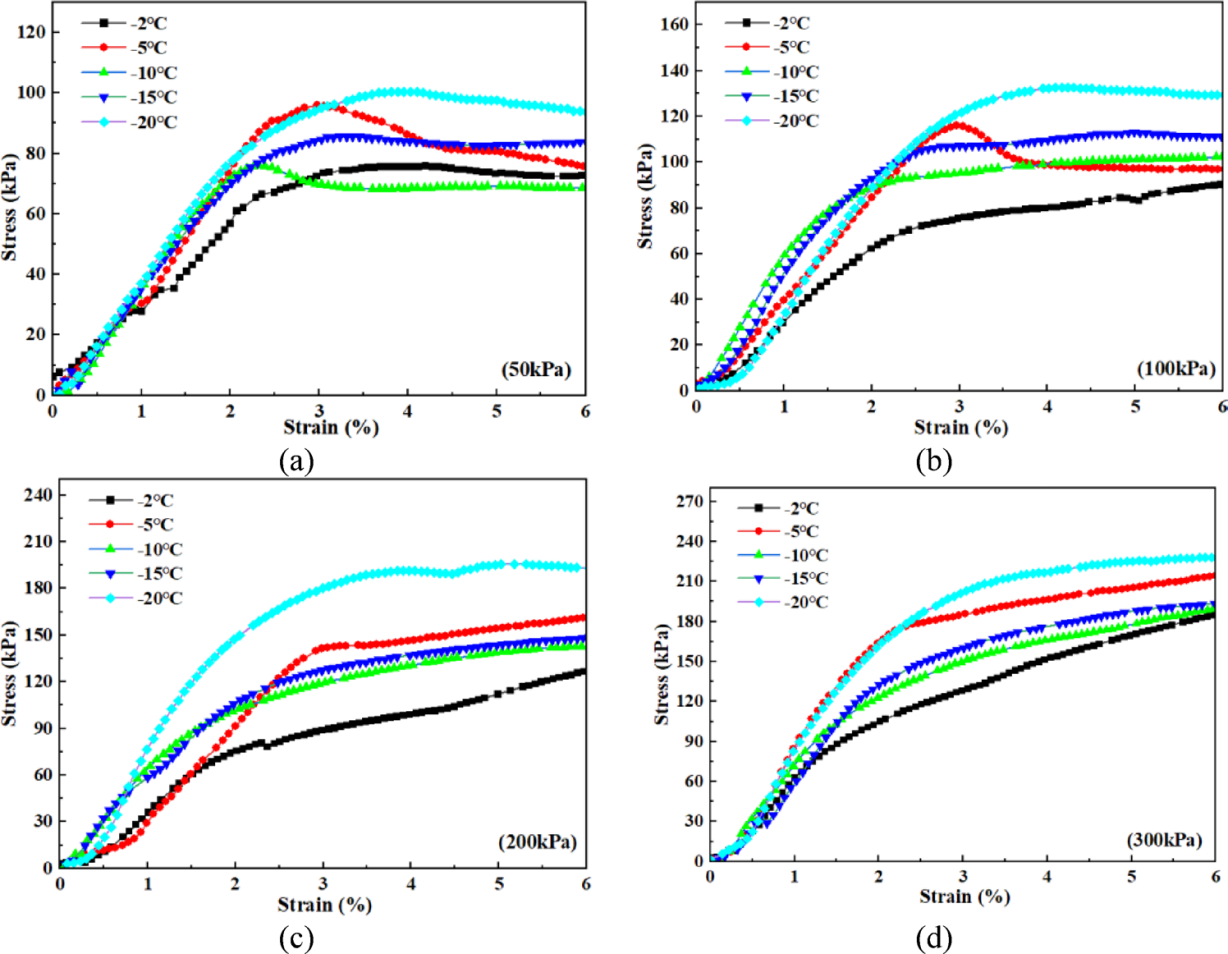
Shear stress-strain curves at different temperatures for 14% moisture content. (**a**) Stress-strain curves at different temperatures under 50 kPa, (**b**) Stress-strain curves at different temperatures under 100 kPa, (**c**) Stress-strain curves at different temperatures under 200 kPa, (**d**) Stress-strain curves at different temperatures under 300 kPa.

**Fig. 7:**
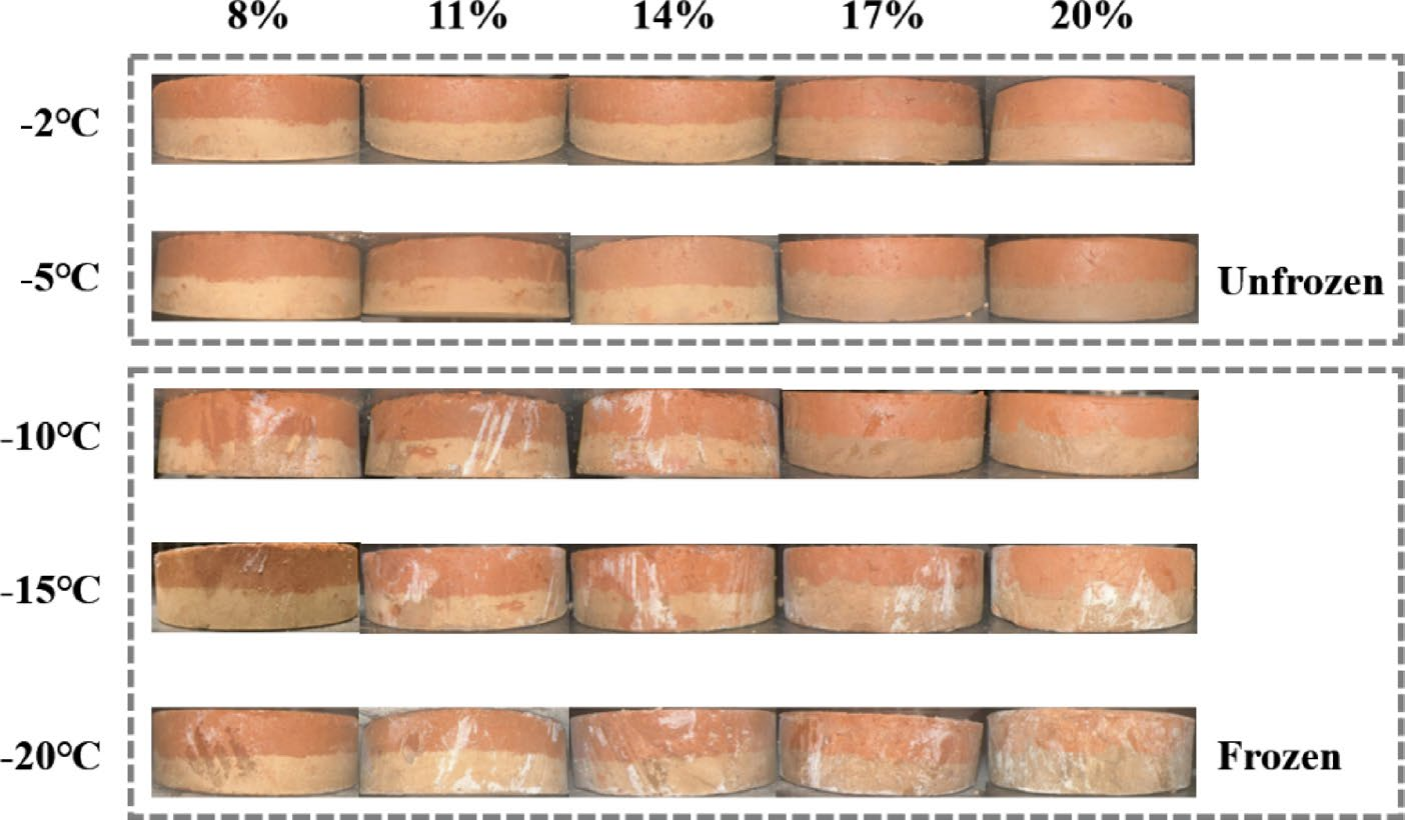
Freezing conditions of the samples.

Figure 6 shows the shear stress-strain curves of the loess-red clay composite at 14% moisture content under different normal stresses (50 kPa, 100 kPa, 200 kPa, 300 kPa) and temperature conditions. When the normal stress is 50 kPa (Fig. 6a), the shear stress–strain curves under different temperature conditions exhibit strain-softening behavior, with weak softening observed at -2 °C, -15 °C and − 20 °C. In the unfrozen stage (-2 °C to -5 °C), as the temperature decreases, the peak strength of the sample increases. At -2 °C, the peak shear stress is 75.6 kPa, 4% higher than the stable strength. When the temperature drops to -5 °C, the peak increases to 90.2 kPa, 22% higher than the residual strength. At this point, compared to -2 °C, the curve exhibits more significant softening characteristics. When the temperature drops to the freezing temperature (-10 °C), the soil begins to freeze (Fig. 7). During this stage, the peak shear stress drops to 75.9 kPa, which is 10% higher than the stable strength, indicating a reduction in the overall strength of the soil. After − 10 °C, the peak shear stress continues to rise as the temperature decreases further: at -15 °C, the peak reaches 85.6 kPa; at -20 °C, the peak reaches 100.3 kPa, and the corresponding strain increases. When the normal stress is 100 kPa (Fig. 6b), the shear stress–strain curve at -5 °C exhibits shear-softening, whereas the curves at other temperatures display weak hardening behavior. As the normal stress increases further to 200 kPa and 300 kPa (Fig. 6c, d), the shear stress-strain curves at all temperatures exhibit shear hardening characteristics. In addition, at all normal stress levels, the multi-segment variation of the peak or stable shear strength in the curve is related to the freezing temperature. Overall, as the normal stress increases, the degree of strain hardening of the sample becomes more pronounced, and the corresponding strength increases. With temperature variation, the multi-stage change observed in the stress–strain curve is related to the freezing temperature. Before freezing, the strain-softening behavior becomes more evident as the temperature decreases; after freezing, the degree of strain hardening increases with decreasing temperature, and the corresponding strength also increases.

### Change of shear strength

**Fig. 8:**
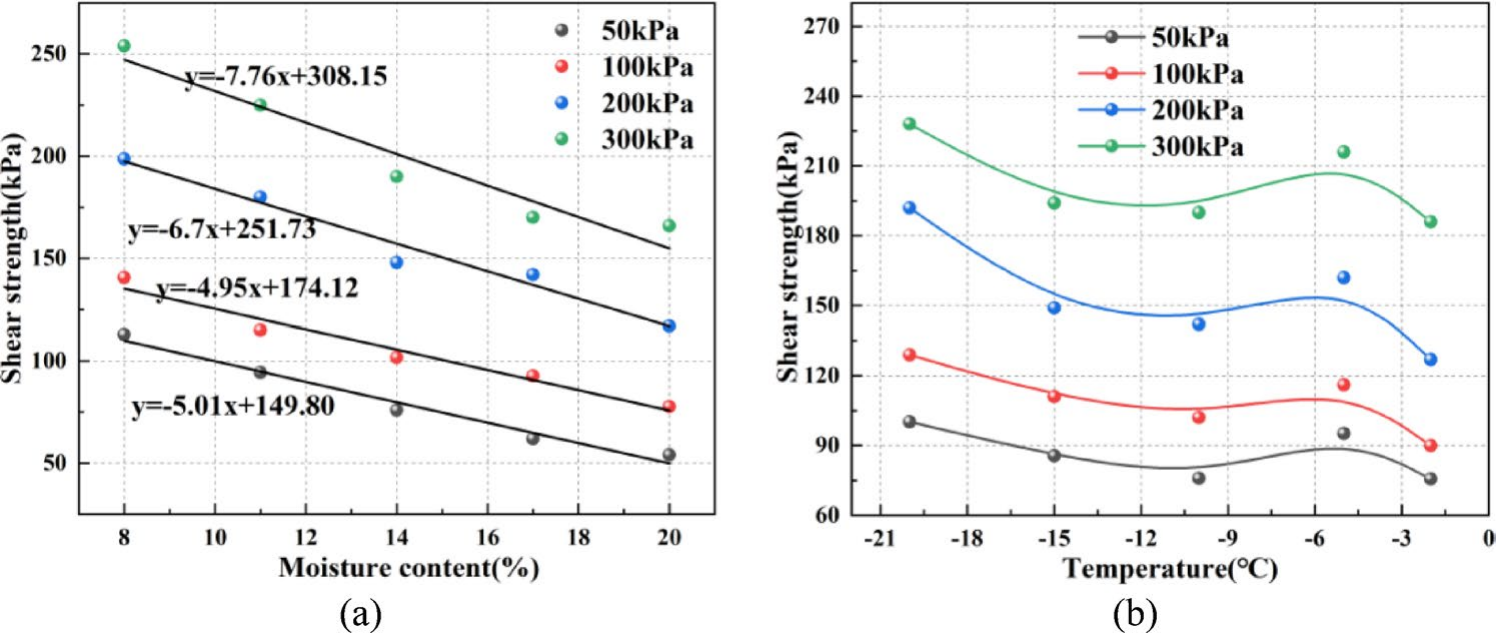
Shear strength of loess–red clay composite interface under different moisture contents and temperatures. (**a**) Shear strength of samples under different moisture contents at-10 °C, (**b**) Shear strength of samples at 14% moisture content under different temperatures.

Figure 8 illustrates the changes of shear strength with moisture content and temperature. Figure 8a presents the trend of shear strength under different normal stresses at -10 °C as the moisture content increases. Overall, under different normal stresses, the shear strength decreases approximately linearly with increasing moisture content. Moreover, the higher the normal stress, the faster the shear strength decreases, indicating stronger water sensitivity of the soil and a more pronounced softening effect of water. For example, at a normal stress of 100 kPa, the shear strength is 140.7 kPa at 8% moisture content. When the moisture content increases to 11%, the shear strength drops to 115.3 kPa, a decrease of 18.1%. As the moisture content further increases to 14%, the shear strength declines to 101.7 kPa, a 11.5% decrease. When the moisture content reaches 17% and 20%, the shear strength decreases by 8.9% and 16.2%, respectively. Figure 8b shows the variation trend of shear strength with temperature under different normal stresses at a moisture content of 14%. The results indicate that under all normal stress conditions, shear strength follows a three-stage trend of “first increasing, then decreasing, and increasing again” as the temperature decreases. For instance, at a normal stress of 200 kPa, the shear strength is 127.3 kPa at -2 °C. As the temperature decreases to -5 °C, the shear strength increases to 162.4 kPa, a rise of 27.6%. When the temperature further drops to the freezing temperature of -10 °C, the sample begins to freeze, causing the shear strength to decrease slightly to 142.9 kPa, a 12.3% drop. However, as the temperature continues to decline and the freezing degree intensifies, the shear strength gradually increases again, reaching 149.6 kPa at -15 °C and 192.3 kPa at -20 °C, with an overall increase of 51.1% during the freezing process. These results suggest that the variation of shear strength with temperature is closely related to the freezing temperature.

**Fig. 9:**
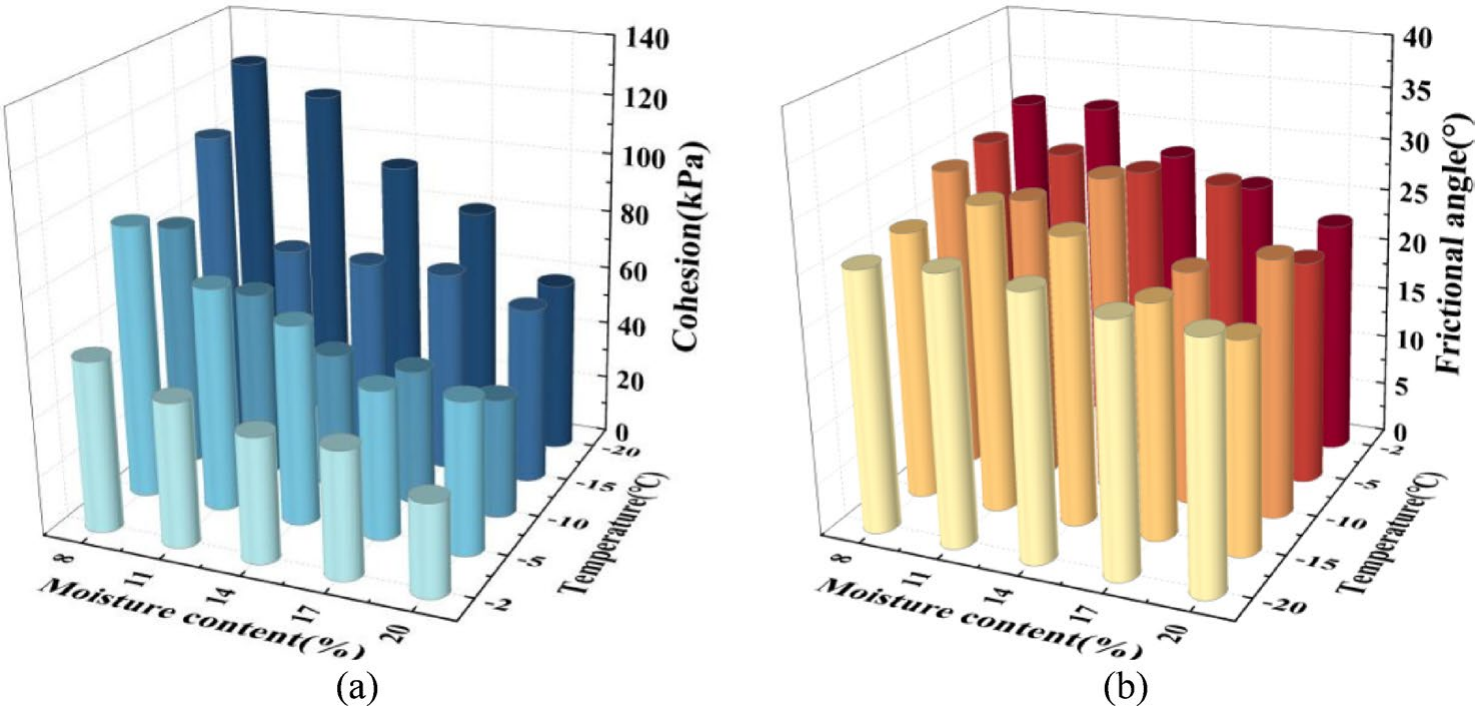
Shear strength parameters of loess–red clay composite interface under different conditions. (**a**) Cohesion of shear samples under different conditions, (**b**) Friction angle of shear samples under different conditions.

Figure 9 illustrates the variations in shear strength parameters, including cohesion and friction angle, with moisture content and temperature. Figure 9a presents the changes in cohesion under different temperature and moisture content conditions. The results show that cohesion decreases with increasing moisture content. For example, at -20 °C, the cohesion of the sample is 125.3 kPa at 8% moisture content, decreasing to 115 kPa at 11%. As the moisture content further increases to 14%, 17%, and 20%, cohesion declines to 93.4 kPa, 80.5 kPa, and 58.5 kPa, respectively, resulting in an overall decrease of 53.3%. Under temperatures ranging from − 2 °C to -15 °C, the reduction in cohesion is 46.5%, 45.1%, 51%, and 35%, respectively, indicating a significant decrease in cohesion with increasing moisture content across all temperature conditions. Additionally, cohesion exhibits a three-stage variation with decreasing temperature, showing an initial increase, followed by a decline, and then another increase. For instance, at 8% moisture content, cohesion rises from 58.5 kPa at -2 °C to 93.3 kPa at -5 °C, then decreases to 83.5 kPa at -10 °C, before recovering to 125.3 kPa at -20 °C. This trend is observed across different moisture content conditions, suggesting that the variation in cohesion with temperature and moisture content is consistent with the changes in shear strength. Figure 9b displays the changes of the friction angle under different temperature and moisture content conditions. The results indicate that the friction angle follows a “first increasing, then decreasing” trend with increasing moisture content. For example, at -2 °C, the friction angle is 31.7° at 8% moisture content and increases slightly to 31.9° at 11%. However, as the moisture content further increases to 14%, 17%, and 20%, the friction angle decreases to 27.9°, 25.6°, and 22.7°, respectively. This trend is observed at other temperature conditions as well, suggesting a nonlinear variation of the friction angle with moisture content. However, under temperature variations, the friction angle does not exhibit a consistent change pattern.

## Discussion and analysis

Shear strength of soil refers to the maximum resistance a soil can withstand under shear force, reflecting its ability to resist shear sliding^30,31^. It is primarily affected by factors such as soil type, moisture content, dry density, structure, stress conditions, temperature, and shear rate^32–39^. In direct shear tests, the shear failure process of soil involves two main aspects of energy consumption: overcoming the cementation force between soil particles and the additional energy required for rearranging the particles after shear. As the shear force increases, the interaction between particles at the loess–red clay interface is dominated by static friction, with no sliding or failure occurring, indicating a relatively stable internal soil structure. When the shear stress reaches its peak, the cementation between soil particles is disrupted^40,41^, leading to shear fracture along the loess–red clay interface. After the peak, the shear force is primarily consumed in overcoming the re-cementation and sliding friction between the loess and red clay layers. The direct shear tests indicate that moisture content and temperature significantly affect the shear strength of the loess-red clay composite interface, which may be closely related to the heterogeneous interface, soil structure characteristics, soil moisture migration, and water-ice phase transition evolution. The influence mechanism of moisture content, temperature, and their coupling effects on shear strength are preliminarily discussed in this section, to better understand the deformation and failure process of the loess-red clay interface, as well as the sliding-induced disaster characteristics.

### Influence of moisture content on soil strength

**Fig. 10:**
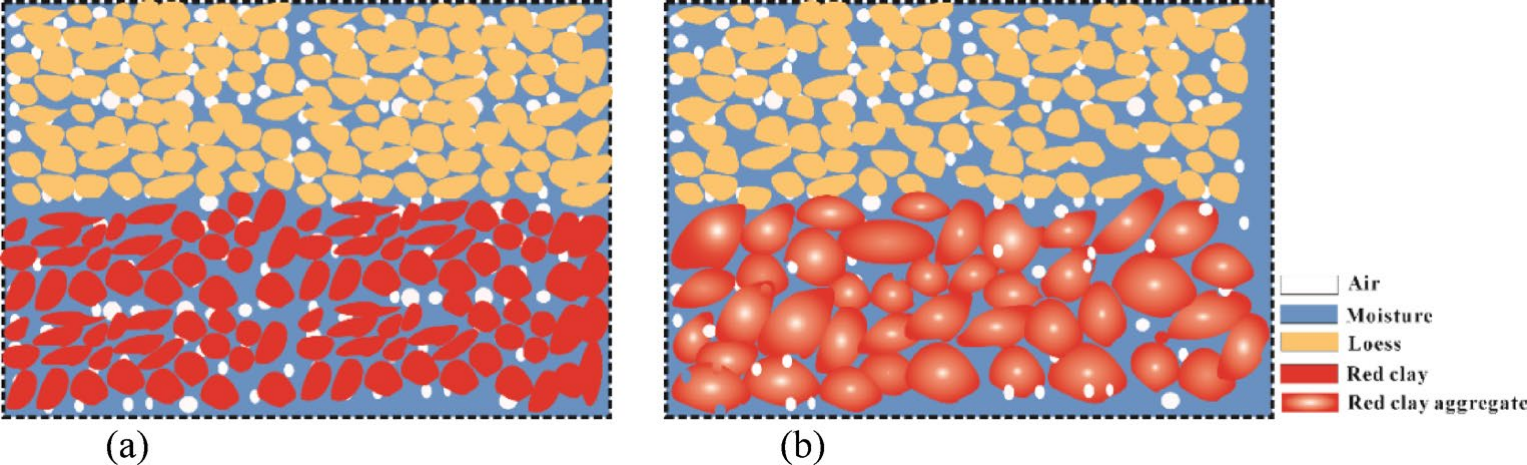
Soil conditions at different moisture contents. (**a**)Soil conditions at low moisture content, (**b**)Soil conditions at high moisture content.

This study found that the initial moisture content affects the shear strength of loess–red clay composite interface mainly in terms of cohesion (Figs. 8 and 9). In this experiment, during the unfrozen stage (–2 °C to − 5 °C), at 8% moisture content (mainly bound water), the soil particles are closely packed, with small relative distances between particles and strong interparticle bonding, resulting in higher cohesion^30^. As the moisture content increases to 14%, the loess and red clay in the sample darken in color, indicating that water fills soil pores (mainly small pores). Meanwhile, hydration occurs in the red clay, leading to the dissolution of minerals and organic matter, forming gel-like substances. These substances enhance adsorption between soil particles, promoting the formation of aggregates^42,43^. Consequently, the red clay layer transitions from a continuous mass to a collection of aggregates, reducing the contact area between the red clay and loess layers and lowering the cohesion. With further increases in moisture content, capillary water causes greater spacing between soil particles, reducing the interparticle interaction forces and weakening the cementation between particles^28,41,44–46^, leading to a further reduction in cohesion at the interface. Additionally, more aggregates begin to appear in the red clay, intensifying the heterogeneous double-layer structure of the sample (Fig. 10). When the moisture content reaches 20%, water fully fills most of the soil pores, and the red clay layer consists of irregularly sized aggregates. Both the loess and red clay layers experience a significant reduction in strength, further decreasing the cohesion at the interface. When the freezing temperature falls below the freezing point (–10 °C to − 20 °C), the moisture in the specimen may not be completely frozen, and the proportion of unfrozen water may even increase with higher moisture content^47^. The softening effect of unfrozen water is stronger than the strengthening effect provided by the additional ice, resulting in a reduction in shear strength. In addition, the loess–red clay composite is subjected to vertical pressure during the shear process, leading to pressure-melting phenomena^48,49^. Under higher pressures, pore ice may partially melt into liquid water, weakening the structural contribution of ice and further increasing the amount of unfrozen water. Consequently, the shear strength continues to decrease as the moisture content rises. It should be noted that the effect of moisture content on the friction between soil particles is relatively minor. When moisture content is high (> 14%), the formation of aggregates in red clay (particle roundness increases, contact points decrease) and the lubricating effect of water on soil particles (thicker adsorbed water film, more particle debris) result in a slight decrease in the internal friction angle^30,41^.

### Influence of temperature on soil shear strength

**Fig. 11:**
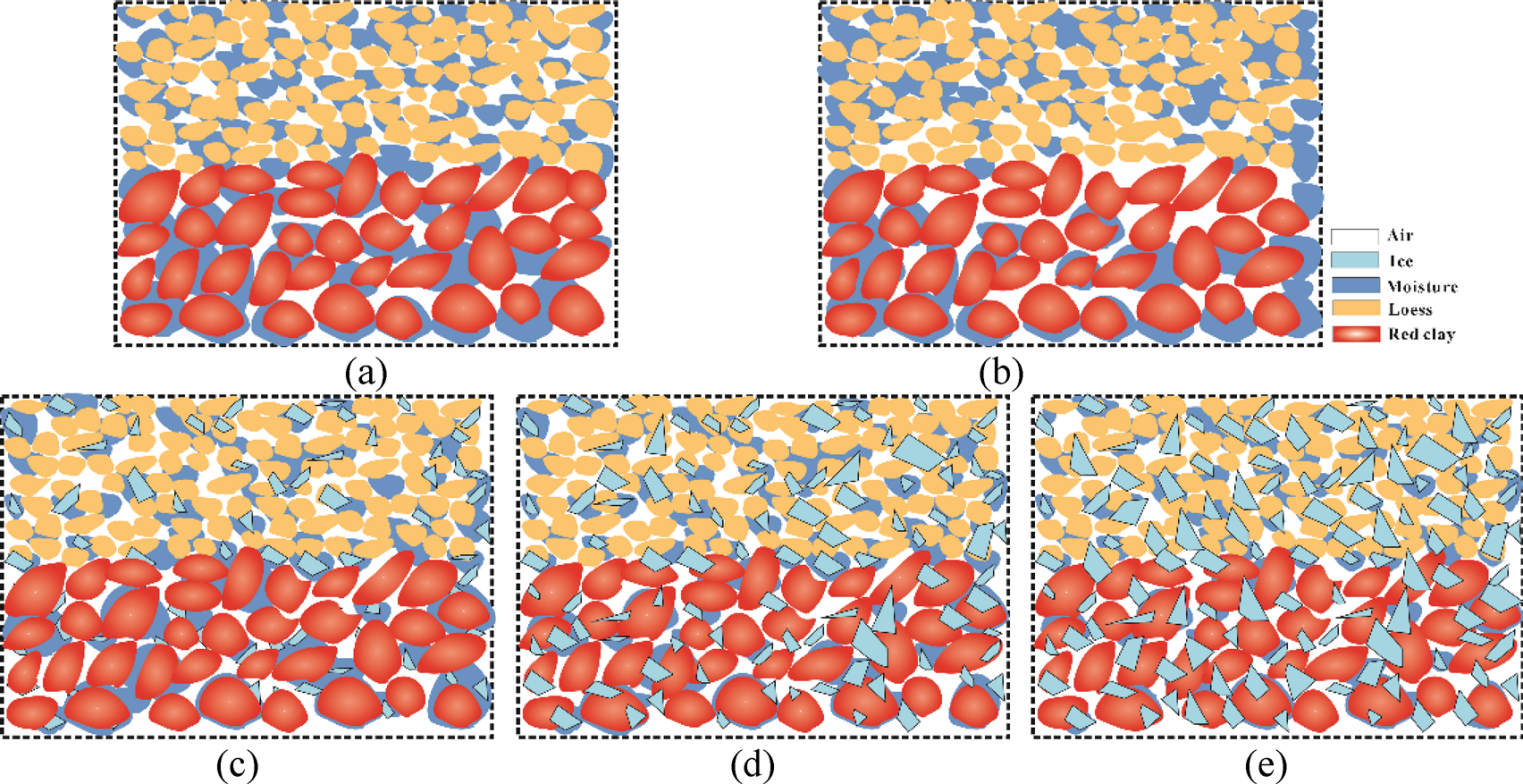
Soil conditions at different temperatures. (**a**)Soil conditions at -2℃, (**b**)Soil conditions at -5℃, (**c**) Soil conditions at -10℃, (**d**) Soil conditions at -15℃, (**e**) Soil conditions at -20℃.

Variations in pore structure and moisture distribution at different temperatures significantly affect the shear strength of soils^50^. In this study, it was found that with decreasing freezing temperature, the shear strength and cohesion of the soil exhibited a three-stage trend of “increase–decrease–increase”, while the internal friction angle was not sensitive to temperature (Figs. 8 and 9). The internal conditions of the specimens at different temperatures are shown in Fig. 11. In the unfrozen stage (–2 °C to − 5 °C), the temperature has not yet reached the freezing point, and the soil remains unfrozen^51,52^. Since the surface temperature of the specimen was lower than its interior, internal water migrated toward the colder surface layer^53,54^. This migration reduced the moisture content at the loess–red clay interface, thereby increasing cohesion and enhancing the shear strength of the composite. When the temperature dropped to the freezing temperature (–10 °C), ice formation began within the soil. The freezing process caused crystallization of pore water, and unfrozen water from the exterior migrated inward toward the growing ice front^55–57^. This inward water migration increased the moisture content at the interface and within the soil, weakening the cementation between the soil particles and resulting in a decrease in shear strength. As the temperature continued to decrease (–10 °C to − 20 °C), unfrozen water within the pores of the loess and red clay gradually transformed into ice crystals. Since ice cementation has greater strength than water (typically outweighing the cohesion of soil particles), the freezing process enhanced the physical bonding between soil particles within the aggregates. Meanwhile, the reduction in unfrozen water further increased the cohesion of the soil^58-60^. Ultimately, at − 20 °C, the soil reached its maximum shear strength. For the internal friction angle, the structural framework of soil after freezing remain relatively stable during shearing, with minimal changes in particle interlocking friction, making the internal friction angle typically insensitive to temperature^61^.

### Coupling effect of moisture and temperature on shear strength

**Fig. 12:**
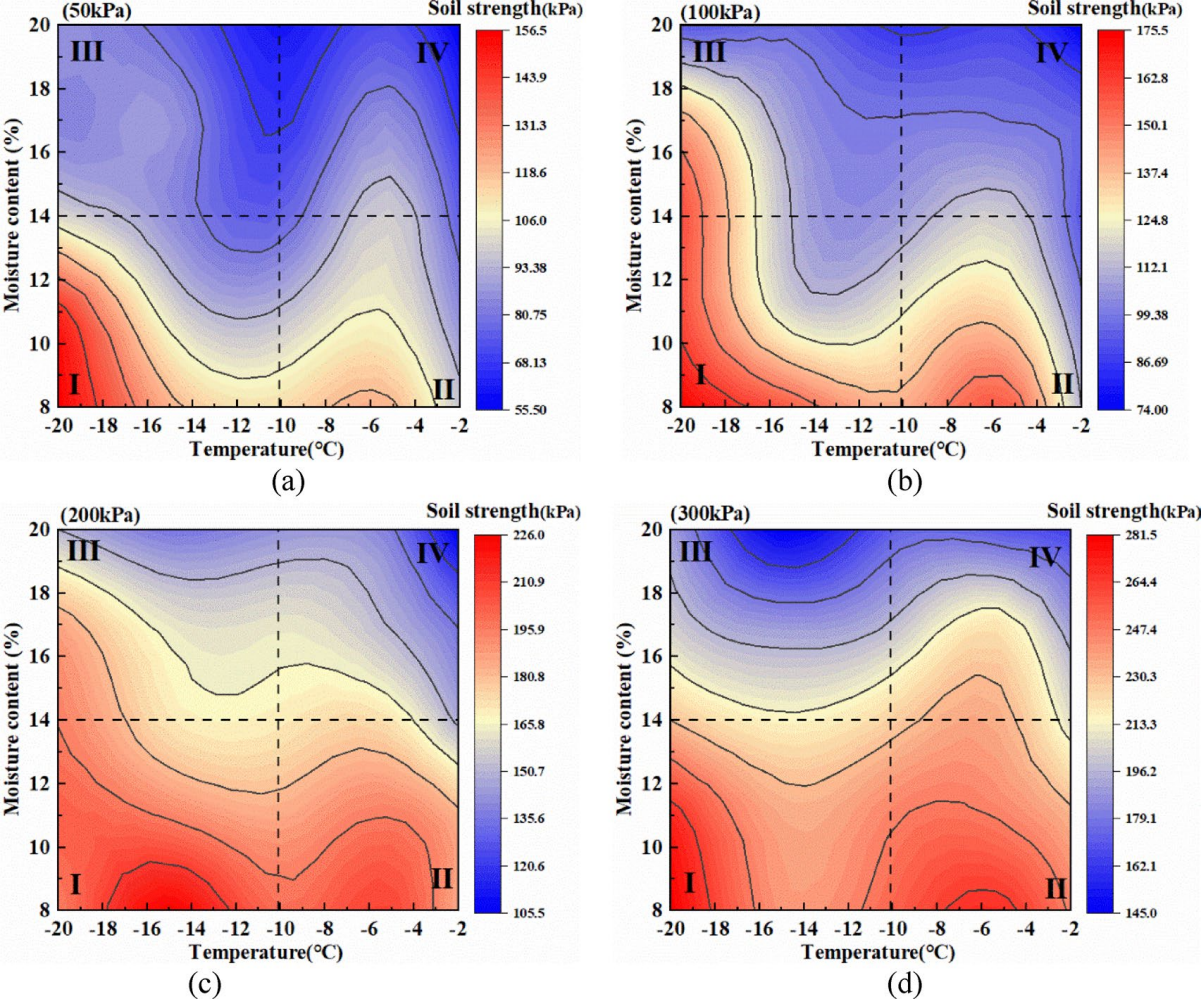
Changes in shear strength with moisture content and temperature under different normal stresses. (**a**) Soil strength at 50 kPa, (**b**) Soil strength at 100 kPa, (**c**) Soil strength at 200 kPa, (**d**) Soil strength at 300 kPa.

The shear strength of the loess–red clay composite is influenced by both moisture and temperature. Moisture determines particle cohesion and dispersion, while temperature affects moisture migration and the consolidation process after freezing. As moisture content increases and temperature decreases, the location, type, phase, and content of moisture within the soil continuously change. This study found that moisture has a more significant impact on shear strength than temperature. Figure 12 illustrates the changes in shear strength of the samples with moisture content and temperature under different normal stresses. Taking 50 kPa (Fig. 12a) as an example, the shear strength can be categorized into four levels based on a freezing temperature of -10 °C and a critical moisture content of 14%, at which red clay begins to form aggregates. The highest strength is observed in Region I (-20 °C to -10 °C, 8% to 14%) with an average of 110.6 kPa, followed by Region II (-10 °C to -2 °C, 8% to 14%) with an average of 96.1 kPa. Region III (-20 °C to -10 °C, 14% to 20%) has an average strength of 80.5 kPa, while Region IV (-10 °C to -2 °C, 14% to 20%) exhibits the lowest value at 73.1 kPa. A similar trend is observed for normal stresses ranging from 100 kPa to 300 kPa (Fig. 12b, c,d), and this trend becomes more pronounced with increasing normal stress. In Regions II and III, temperature and moisture content have opposite effects on shear strength. Region II has a lower moisture content than Region III, whereas Region III contains more consolidated ice. However, the average shear strength of Region II is higher than that of Region III because the strengthening effect of ice consolidation in Region III is weaker than the softening effect of moisture on the soil, leading to a lower overall shear strength. Additionally, at (-5 °C, 8%), a peak shear strength is observed. This occurs because, at -5 °C, moisture migrates to the surface, reducing the moisture content at the loess–red clay interface, which enhances shear strength. As the temperature continues to decrease, moisture migrates inward, leading to a reduction in strength. Simultaneously, the increasing moisture content softens the soil, further reducing shear strength.

## Conclusion

This study focused on the loess–red clay composite to investigate the variation in shear behavior of interface specimens with different moisture contents under low-temperature conditions. The results were discussed based on the heterogeneous interface, soil structure, and the migration and occurrence evolution characteristics (location, type, phase, and content) of soil moisture in response to freezing temperatures. The main conclusions are as follows:

1. The shear strength of loess–red clay specimens generally decreases with increasing moisture content. Before the freezing point, higher water content fills the soil pores, increases particle spacing, weakens cementation, and enhances the structural heterogeneity between the two soil layers, leading to a reduction in strength. After the freezing point, as the water content increases, the proportion of unfrozen water in the soil continues to rise. Its softening effect on shear strength outweighs the strengthening contribution of consolidated ice, resulting in a continued decrease in shear strength under frozen conditions.

2. The shear strength of the specimens exhibits a three-stage trend with decreasing temperature: initial increase, subsequent decrease, and final increase. In the unfrozen stage (–2 °C to − 5 °C), moisture migrates from the interior toward the colder surface, increasing the interface shear strength. When the temperature drops to the freezing point (–10 °C), freezing initiates, and unfrozen water from the exterior migrates inward toward the growing ice front, weakening the cementation and reducing the shear strength. As the temperature continues to decrease (below − 10 °C), unfrozen water within the soil gradually transforms into consolidated ice. The reduction in free water and the increase in ice content enhance particle bonding and cementation, resulting in a rise in shear strength.

3. Moisture content and temperature exhibit a coupling effect on the shear strength of the loess–red clay composite, with moisture content being the dominant factor. Based on the freezing temperature (–10 °C) and the moisture content threshold for aggregate formation (14%), the shear strength variation can be divided into four zones. In each zone, the shear strength is negatively correlated with moisture content, while no consistent pattern is observed with ice content, indicating that water plays a key role in the coupling mechanism affecting soil strength.

This study provides a preliminary exploration of the shear behavior of loess–red clay interfaces under low-temperature conditions. The findings not only offer valuable data for stability monitoring and geotechnical engineering applications in both seasonally and permanently frozen regions of the Loess Plateau, but also provide forward-looking guidance for the prevention and control of loess geological disasters and ecological environment protection in special environments. However, this study does not account for the microstructural differences between undisturbed and remolded soils. Future work will employ a large-scale visual direct shear apparatus to systematically investigate the full evolution of shear deformation and multi-source responses of loess–red clay heterogeneous interfaces under complex environmental conditions, with particular emphasis on microstructural characteristics and the ice–water phase transition at the interface. This research is currently underway.

## Data Availability

The datasets used and/or analysed during the current study available from the corresponding author on reasonable request.

## References

[1] Peng, J. B. et al. Distribution and genetic types of loess landslides in China. *J. Asian Earth Sci.* **170**, 329–350. 10.1016/j.jseaes.2018.11.015 (2019).

[2] Lawrence, J. F. et al. Observations on the hipparion red clays of the loess plateau. *Vertebrata Palasiatica* **49**(3), 275–284.

[3] Qu, Y. X., Zhang, Y. S. & Qin, Z. M. Hipparion laterite and landslide hazards on loess plateau of Northwestern China. *J. Eng. Geol.* **7** (3), 257–265 (1999).

[4] Wang, Q. Q., Li, W. P., Guo, Y. H., Yang, Y. R. & Fan, K. F. Geological and geotechnical characteristics of N2 laterite in Northwestern China. *Quatern Int.* **519**, 263–273. 10.1016/j.quaint.2019.02.009 (2019).

[5] Zhang, M. & Liu, J. Controlling factors of loess landslides in Western China. *Environ. Earth Sci.* **59** (8), 1671–1680 (2010).

[6] Chen, B. et al. Study of the low-temperature responses of the resistivity and P-wave velocity of the loess-red clay composite. *Bull. Eng. Geol. Environ.* **84** (3), 153. 10.1007/s10064-025-04151-z (2025).

[7] Shi, J. et al. Neogene clay and its relation to landslides in the Southwestern loess Plateau, China. *Environ. Earth Sci.* **77** (5), 204. 10.1007/s12665-018-7350-5 (2018).

[8] Xie, C. W., Gough, W. A., Tam, A., Zhao, L. & Wu, T. H. Characteristics and persistence of relict high-altitude permafrost on Mahan Mountain, loess Plateau, China. *Permafr. Periglac.* **24** (3), 200–209. 10.1002/ppp.1776 (2013).

[9] Jin, H. J. et al. Quaternary permafrost in china: framework and discussions. *Quaternary* **3** (4), 32. 10.3390/quat3040032 (2020). (ESCI).

[10] Wang, X. Q. & Chen, R. S. Freezing and thawing characteristics of seasonally frozen ground across China. *Geoderma* **448**, 116966. 10.1016/j.geoderma.2024.116966 (2024).

[11] Li, B., Feng, Z. & Wang, W. P. Characteristics of the Sanmen Formation clays and their relationship with loess landslides in the Guanzhong area, Shaanxi, China. *Arab. J. Geosci.* **8** (10), 7831–7843 10.1007/s12517-015-1822-7 (2015).

[12] Derbyshire, E. Geological hazards in loess terrain, with particular reference to the loess regions of China. *Earth Sci. Rev.* **54** (1–3), 231–260 10.1016/S0012-8252(01)00050-2 (2011).

[13] Wang, N. Q. & Yao, Y. Characteristics and mechanism of landslides in loess during freezing and thawing period in seasonally frozen ground regions. *J. Disaster Prev. Mitigation Eng.* **28** (2), 163–166. 10.3969/j.issn.1672-2132.2008.02.006 (2008).

[14] Shi, J. S. et al. Analysis of the causes of large-scale loess landslides in Baoji, China. *Geomorphology* **264**, 109–117. 10.1016/j.geomorph.2016.04.013 (2016).

[15] Li, B., Wu, S. R., Shi, J. S. & Feng, Z. Engineering geological properties and hazard effects of hipparion laterite in Baoji, Shaanxi Province. *Geol. Bull. China*. **32** (12), 1918–1924 (2013).

[16] Wen, T. et al. Shear behavior of gravel-block soil of the Qinghai-Tibet plateau based on large-scale direct shear test and numerical simulation. *Bull. Eng. Geol. Environ.* **84** (6), 294. 10.1007/s10064-025-04346-4 (2025).

[17] Yang, X. Y., Wang, Y. C. & Sun, Z. J. The shearing anisotropy characteristics on the interface of loess with bedrock. *Bull. Eng. Geol. Environ.* **79** (10), 5205–5212. 10.1007/s10064-020-01887-8 (2020).

[18] He, P. F. et al. Experimental study on the effect of freeze-thaw cycles on the shear characteristics of frozen soil-composite geotextile interface. *Case Stud. Therm. Eng.* **54**, 104011. 10.1016/j.csite.2024.104011 (2024).

[19] Ying, M. J., Liu, F. Y., Wang, J., Wang, C. L. & Li, M. F. Coupling effects of particle shape and Cyclic shear history on shear properties of coarse-grained soil-geogrid interface. *Transp. Geotech.* **27**, 100504. 10.1016/j.trgeo.2020.100504 (2021).

[20] Jitsangiam, P. et al. Characterization of a soil-rough structure interface using direct shear tests with varying Cyclic amplitude and loading sequences under a large Cyclic testing cycle condition. *Acta Geotech.* **17** (5), 1829–1845. 10.1007/s11440-021-01289-4 (2022).

[21] Anda, R., Wang, L., Ying, M. J. & Huang, Y. T. Analysis of shear characteristics of recycled concrete Aggregate-Geogrid interface. *J. Mater. Civil Eng.* **35** (7), 12. 10.1061/JMCEE7.MTENG-15365 (2023).

[22] Liu, J. K., Lv, P., Cui, Y. H. & Liu, J. Y. Experimental study on direct shear behavior of frozen soil-concrete interface. *Cold Reg. Sci. Technol.* **104**, 1–6. 10.1016/j.coldregions.2014.04.007 (2014).

[23] Cen, D. F., Huang, D. & Ren, F. Shear deformation and strength of the interphase between the soil-rock mixture and the benched bedrock slope surface. *Acta Geotech.* **12** (2), 391–413. 10.1007/s11440-016-0468-2 (2017).

[24] Li, D. J., Shi, C., Ruan, H. N. & Li, B. Y. Shear characteristics of soil-concrete structure interaction interfaces. *Appl. Sci. -Basel*. **12** (18), 9145. 10.3390/app12189145 (2022).

[25] Xin, Y. et al. Study on the direct shear failure characteristics of the loess-Hipparion red clay interface. *J. Eng. Geol.* **33** (04), 1250–1261. 10.13544/j.cnki.jeg.2023-0228 (2025).

[26] Zhu, Y. B., Han, Y. T., Lan, H. X., Miao, S. S. & Li, W. J. Study on the influence of contact angle on the shear mechanical properties of the loess-Hipparion red clay interface. *J. Eng. Geol.* **29** (3), 879–890. 10.13544/j.cnki.jeg.2021-0041 (2021).

[27] Zhu, Y. B., Miao, S. S., Li, H. F., Han, Y. T. & Lan, H. X. An empirical shear model of interface between the loess and hipparion red clay in a loess landslide. *Front. Earth Sci.* **9**, 806832. 10.3389/feart.2021.806832 (2022).

[28] Lian, B. Q., Wang, X. G., Liu, K., Hu, S. & Feng, X. A mechanical insight into the triggering mechanism of frequently occurred landslides along the contact between loess and red clay. *Sci. Rep.* **11** (1), 17556. 10.1038/s41598-021-96384-7 (2021). 34475420 10.1038/s41598-021-96384-7 PMC8413338

[29] Wen, B. P., Wang, S. J. & Wang, E. Z. Deformation characteristics of loess landslide along the contact between loess and neocene red mudstone. *Acta Geol. Sinica-Engl Ed.* **79** (1), 139–151 (2005).

[30] Gu, T. F. et al. Experimental study of the shear strength of soil from the Heifangtai platform of the loess plateau of China. *J. Soils Sediments*. **19** (10), 3463–3475. 10.1007/s11368-019-02303-9 (2019).

[31] Xu, L. et al. Landslides in a loess platform, north-west China. *Landslides* **11** (6), 993–1005. 10.1007/s10346-013-0445-x (2014).

[32] Zhang, F. Y., Wang, G. H., Kamai, T. & Chen, W. W. Effect of pore-water chemistry on undrained shear behaviour of saturated loess. *J. Eng. Geol. Hydrogeol.* **47** (3), 201–209. 10.1144/qjegh2013-085 (2014).

[33] Lian, B. Q., Peng, J. B., Wang, X. G. & Huang, Q. B. Moisture content effect on the ring shear characteristics of slip zone loess at high shearing rates. *Bull. Eng. Geol. Environ.* **79** (2), 999–1008. 10.1007/s10064-019-01597-w (2020).

[34] Bao, W. X., Wang, H. B., Lai, H. P. & Chen, R. Experimental study on strength characteristics and internal mineral changes of Lime-stabilized loess under High-Temperature. *Constr. Build. Mater.* **351**, 128945. 10.1016/j.conbuildmat (2022).

[35] Yazdani, S., Helwany, S. & Olgun, G. Influence of temperature on soil–pile interface shear strength. *Geomech. Energy Envir*. **18**, 69–78. 10.1016/j.gete.2018.08.001 (2019).

[36] Xu, J. et al. Influence of dry-wet cycles on the strength behavior of basalt-fiber reinforced loess. *Eng. Geol.* **302**, 106645. 10.1016/j.enggeo.2022.106645 (2022).

[37] Yang, Z. et al. Study on influencing factors and prediction model of strength and compression index of sandy silt on bank under freeze-thaw cycles. *Sci. Rep.* **15** (1), 1402. 10.1038/s41598-025-85789-3 (2025). 39789285 10.1038/s41598-025-85789-3 PMC11718174

[38] Wei, Y. Z., Yao, Z. H., Chong, X. L., Zhang, J. H. & Zhang, J. Microstructure of unsaturated loess and its influence on strength characteristics. *Sci. Rep.* **12** (1), 1502. 10.1038/s41598-022-05464-9 (2022). 35087133 10.1038/s41598-022-05464-9 PMC8795260

[39] Chen, Y., Zhang, R. Y., Zi, J. J., Han, J. Y. & Liu, K. W. Evaluation of the treatment variables on the shear strength of loess treated by microbial induced carbonate precipitation. *J. Mt. Sci.* **22** (3), 1075–1086. 10.1007/s11629-024-9100-3 (2025).

[40] Wei, T. T. et al. Quantification of the spatial-temporal evolution of loess microstructure from the Dongzhi tableland during shearing. *Eng. Geol.* **323**, 107213. 10.1016/j.enggeo.2023.107213 (2023).

[41] Nan, J. J. et al. Shear behavior and microstructural variation in loess from the yan’an area, China. *Eng. Geol.* **280**, 105964. 10.1016/j.enggeo.2020.105964 (2021).

[42] Paul, B. K. et al. Medium-term impact of tillage and residue management on soil aggregate stability, soil carbon and crop productivity. *Agric. Ecosyst. Environ.* **164**, 14–22 10.1016/j.agee.2012.10.003 (2013).

[43] Yu, Z. H., Zhang, J. B., Zhang, C. Z., Xin, X. L. & Li, H. The coupling effects of soil organic matter and particle interaction forces on soil aggregate stability. *Soil. Till Res.* **174**, 251–260. 10.1016/j.still.2017.08.004 (2017).

[44] Li, X. A. et al. Characterization of the mechanisms underlying loess collapsibility for land-creation project in Shaanxi Province, China-a study from a micro perspective. *Eng. Geol.* **249**, 77–88. 10.1016/j.enggeo.2018.12.024 (2019).

[45] Muñoz-Castelblanco, J., Pereira, J. M., Delage, P. & Cui, Y. J. Some aspects of the compression and collapse behaviour of an unsaturated natural loess. *Géotech Lett.* **1**, 17–22. 10.1680/geolett.11.00003 (2011).

[46] Yates, K., Fenton, C. H. & Bell, D. H. A review of the geotechnical characteristics of loess and loess-derived soils from Canterbury, South Island, new Zealand. *Eng. Geol.* **236**, 11–21. 10.1016/j.enggeo.2017.08.001 (2018).

[47] Huang, W. J., Mao, X. S., Wu, Q. & Chen, L. L. Experimental study on shear characteristics of the silty clay soil-ice interface. *Sci. Rep.* **12** (1), 19687. 10.1038/s41598-022-23086-z (2022). 36385115 10.1038/s41598-022-23086-z PMC9669033

[48] Jia, H. L. et al. A resistivity-based study on the pressure melting of pore ice in frozen gravel soil. *Rock. Soil. Mech.* **45** (8), 2221–2231. 10.26599/rsm.2024.9436535 (2024).

[49] Schwegler, E., Sharma, M., Gygi, F. & Galli, G. Melting of ice under pressure. *Proc. Natl. Acad. Sci.* **105** (39), 14779–14783. 10.1073/pnas.0808137105 (2008). 18809909 10.1073/pnas.0808137105 PMC2547417

[50] Zhao, L. Q., Peng, J. B., Ma, P. H., Leng, Y. Q. & Ma, Z. Microstructure response to shear strength deterioration in loess after freeze-thaw cycles. *Eng. Geol.* **323**, 107229. 10.1016/j.enggeo.2023.107229 (2023).

[51] Bing, H. & Ma, W. Laboratory investigation of the freezing point of saline soil. *Cold Reg. Sci. Technol.* **67** (1–2), 79–88. 10.1016/j.coldregions.2011.02.008 (2011).

[52] Shah, R. & Mir, B. A. The freezing point of soils and the factors affecting its depression. In *Advances in Construction Management: Select Proceedings of ACMM 2021* (pp. 157–166) DOI: 10.1007/978-981-16-5839-6_14https://link.springer.com/content/pdf/ (Springer, 2022).

[53] Hoekstra, P. Moisture movement in soils under temperature gradients with the cold-side temperature below freezing. *Water Resour. Res.* **2** (2), 241–250. 10.1029/wr002i002p00241 (1966).

[54] Takashi, T., Ohrai, T., Yamamoto, H. & Okamoto, J. Upper limit of heaving pressure derived by pore-water pressure measurements of partially frozen soil. *Dev. Geotech. Eng.* **28**, 245–257. 10.1016/0013-7952(81)90064-8 (1982).

[55] Qin, B. et al. Study on thermal-hydro-mechanical coupling and stability evolution of loess slope during freeze-thaw process. *Earth Surf. Proc. Land.* **49(6)**, 2010–2026. 10.1002/esp.5812 (2024).

[56] ONeill, K. & Miller, R. D. Exploration of a rigid ice model of Frost heave. *Water Resour. Res.* **21** (3), 281–296. 10.1029/wr021i003p00281 (1985).

[57] Zhang, Y. Z., Wang, T. L., Kou, X. K., Feng, Z. X. & Liu, W. L. Liquid water–vapour migration tracing and characteristics of unsaturated coarse-grained soil in high-speed railway subjected to freezing and different load types. *Constr. Build. Mater.* **283**, 122747. 10.1016/j.conbuildmat.2021.122747 (2021).

[58] Chen, H. E., Guo, H. T., Yuan, X. Q., Chen, Y. T. & Sun, C. Effect of temperature on the strength characteristics of unsaturated silty clay in seasonal frozen region. *KSCE J. Civ. Eng.* **24**, 2610–2620. 10.1007/s12205-020-1974-1 (2020).

[59] Liu, X. Y., Liu, E. L., Zhang, D., Zhang, G. & Song, B. T. Study on strength criterion for frozen soil. *Cold Reg. Sci. Technol.* **161**, 1–20. 10.1016/j.coldregions.2019.02.009 (2019).

[60] Guo, Z. Y. et al. Significance analysis of the factors influencing the strength of the frozen soi-structure interface and their interactions in different phase transition zones. *Case Stud. Therm. Eng.* **50**, 103475. 10.1016/j.csite.2024.105217 (2019).

[61] Wang, W. P. et al. Variation characteristics and influencing factors of loess shear strength in seasonal frozen soil region. *J. Harbin Inst. Technol‌*. **54** (8), 143–150. 10.11918/202104143 (2022).

